# SEMA6D Differentially Regulates Proliferation, Migration, and Invasion of Breast Cell Lines

**DOI:** 10.1021/acsomega.2c00840

**Published:** 2022-04-27

**Authors:** Zehra Elif Gunyuz, Ece Sahi-Ilhan, Cansu Kucukkose, Dogac Ipekgil, Gunes Tok, Gulistan Mese, Engin Ozcivici, Ozden Yalcin-Ozuysal

**Affiliations:** † Department of Molecular Biology and Genetics, Izmir Institute of Technology, 35430 Izmir, Turkey; ‡ Department of Bioengineering, Izmir Institute of Technology, 35430 Izmir, Turkey

## Abstract

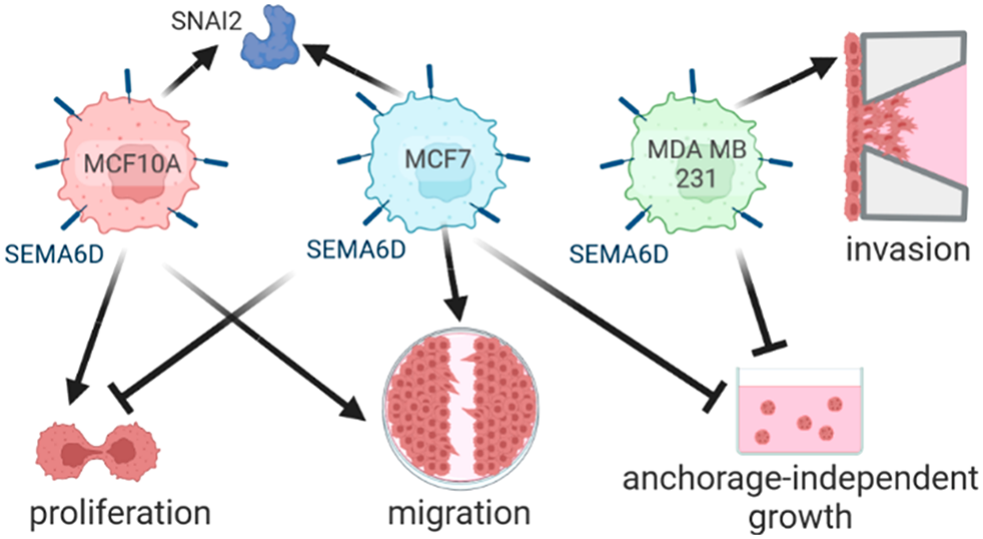

Semaphorin 6D (SEMA6D), a member of the class 6 semaphorin family, is a membrane-associated protein that plays a key role in the development of cardiac and neural tissues. A growing body of evidence suggests that SEMA6D is also involved in tumorigenesis. In breast cancer, high SEMA6D levels are correlated with better survival rates. However, very little is known about the functional significance of SEMA6D in breast tumorigenesis. In the present study, we aimed to investigate the effects of SEMA6D expression on the normal breast cell line MCF10A and the breast cancer cell lines MCF7 and MDA MB 231. We demonstrated that SEMA6D expression increases the proliferation of MCF10A cells, whereas the opposite effect was observed in MCF7 cells. SEMA6D expression induced anchorage-independent growth in both cancer cell lines. Furthermore, migration of MCF10A and MCF7 cells and invasion of MDA MB 231 cells were elevated in response to SEMA6D overexpression. Accordingly, the genes related to epithelial-mesenchymal transition (EMT) were altered by SEMA6D expression in MCF10A and MCF7 cell lines. Finally, we provided evidence that SEMA6D levels were associated with the expression of the cell cycle, EMT, and Notch signaling pathway-related genes in breast cancer patients’ data. We showed for the first time that SEMA6D overexpression has cell-specific effects on the proliferation, migration, and invasion of normal and cancer breast cell lines, which agrees with the gene expression data of clinical samples. This study lays the groundwork for future research into understanding the functional importance of SEMA6D in breast cancer.

## Introduction

Semaphorin 6D (SEMA6D) belongs to class 6 of semaphorins, which constitute a large family of secreted and membrane-bound proteins. Semaphorins were initially identified as regulators of axon guidance during the development of the nervous system by providing repellent or attractant cues. Further studies revealed that semaphorin family members are involved in tissue homeostasis as well as tumorigenesis through the regulation of cell proliferation, cell survival, cell adhesion and migration, immune response regulation, and angiogenesis.^1^

SEMA6D research extensively focused on developmental aspects highlighting its role in axonal extension, cardiac development, neural tube closure, anti-inflammatory macrophage polarization, oligodendrocyte positioning, and crossing of retinal ganglion cells.^2−8^ Beyond its function in developmental processes, accumulating evidence suggests that SEMA6D also plays a part in tumorigenesis. In osteosarcoma, SEMA6D was identified as a candidate oncogene through transposon mutagenesis screening. The functional analysis supported the oncogenic potential of SEMA6D in osteosarcoma, where its overexpression increased soft agar colony formation, cell proliferation, and in vivo tumor formation through ERK phosphorylation.^9^ More recent research also presented evidence for an active role of SEMA6D in the cisplatin resistance mechanism in osteosarcoma.^10^ Likewise, SEMA6D showed an oncogenic behavior in mesothelioma by protecting the cells from apoptosis and increasing soft agar colony formation.^11^ Supporting these findings, in gastric and esophageal cancers, SEMA6D expression was found to be increased in tumors compared to normal tissues.^12^ On the other hand, in lung adenocarcinoma, SEMA6D expression was downregulated, and lower SEMA6D levels were associated with shorter overall survival indicating a tumor suppressor role in the lung.^13^ Altogether, the data suggest a context-dependent role for SEMA6D in tumorigenesis.

Breast cancer is the most commonly diagnosed cancer type and the leading cause of cancer-related deaths in women.^14^ To develop better diagnostic and therapeutic approaches, the fundamental issue is understanding the molecular mechanisms underlying breast cancer. In this context, the role of SEMA6D in breast tumorigenesis remains largely unexplored. What we know is based on the two previous studies that examined breast cancer patient samples.^15,16^ The first study analyzed SEMA6D expression in 1100 samples through The Cancer Genome Atlas (TCGA) database and reported that a high SEMA6D expression is correlated with better survival, which is more significantly pronounced in the triple-negative subgroup.^15^ More recently, single nucleotide variations in SEMA6D were reported in two of the 37 young patients with early onset luminal breast cancer.^16^ Moreover, in line with the previous data, overall survival and relapse-free survival rates were better in the patient group with a higher SEMA6D expression.^16^ Research to date has not yet thoroughly determined the functional significance of SEMA6D expression in breast cancer.

Here, we aimed to analyze cellular and molecular changes in response to SEMA6D overexpression in normal breast and breast cancer cell lines. We showed that although SEMA6D overexpression induced a pro-tumorigenic phenotype in the normal cell line, it negatively affected tumorigenic traits of the cancer cell lines while promoting a more migratory and mesenchymal phenotype in both normal and cancer cell lines. The experimental work we present here provides the first evidence showing that SEMA6D has cell-specific effects in breast cancer, which might reflect on the different clinical outcomes in different breast cancer patient groups.

## Materials and Methods

### Cell Lines and Viral Infections

MCF10A, MCF7, MDA MB 231, and HEK293T cells were obtained from American Type Culture Collection, ATCC. MCF10A cells were cultured in high glucose DMEM-F12 (Gibco, 31330-038) supplemented with 5% horse serum (Gibco, 16050–122), 1% penicillin/streptomycin (Thermo-Fisher Scientific, 15140-122), 20 ng/mg EGF (Sigma, E9644), 0.5 μg/mg hydrocortisone (Sigma, H0888), 100 ng/mL choleratoxin (Sigma, C8052), and 10 μg/mg insulin (Sigma, I1882). MDA MB 231, MCF7, and HEK293T cell lines were maintained in high glucose DMEM (Gibco, 41966-029) supplemented with 10% fetal bovine serum (FBS) (Gibco, 10270106) and 1% penicillin/streptomycin (Gibco, 15140-122). All cell lines were maintained in a humidified incubator with 5% CO_2_ at 37 °C. Overexpression of SEMA6D was performed by a lentiviral expression system. Viruses were prepared, and infections were carried out as explained previously.^17^ pLX304-SEMA6D plasmid was obtained from Dharmacon (OHS6085-213576758) (GenBank reference: BC150253). LacZ in pLX304 (pLX304-LacZ) was a gift from William Hahn (Addgene plasmid no. 42560).^18^ Stable cell lines were generated by 2 μg/mL blasticidin (Santa Cruz, sc-204655A) selection. MCF10A cells that have increased Notch activation upon overexpression of Notch1 intracellular domain (NICD) were described previously.^17^

### RNA Isolation and qRT-PCR

Total RNA was isolated using the Pure-link RNA mini kit (Invitrogen, 12183018A). cDNA was synthesized using the RevertAid first-strand cDNA synthesis kit (Thermo Scientific, K1622). PCR amplification and detection were done on Roche LightCycler 96 Real-Time PCR Detection System using FastStart Essential DNA Green Master (Roche, 06402712001). TATA-box binding protein (TBP) was used as the internal control for normalization. Relative mRNA levels were calculated using delta–delta Ct method. Each experiment is normalized to its own control condition. At least three independent experiments were done. The primer pairs are listed in Table S1.

### Protein Isolation and Western Blot Analysis

Total protein was extracted using RIPA lysis buffer. Conditioned medium (CM) was collected from 3.5 × 10^5^ MCF10A cells, 4 × 10^5^ MDA MB 231, and MCF7 cells seeded on a 6-well plate after 2 days of incubation with serum-free medium. Protein isolation from CM was done by methanol/chloroform precipitation as described previously.^19^ The amount of protein was quantified with Bradford Assay. The total amount of protein of 60 μg from cell lysates and 20 μg from CM were separated via SDS-gel and transferred onto polyvinylidene fluoride (PVDF) membranes. After blocking with 5% skim milk, the membranes were incubated with the following antibodies: anti-β-actin (Abcam, ab75186, 1:2000), anti-*N*-cadherin (Cell Signaling Technology, 13116T, 1:1000), anti-SNAI2 (Cell Signaling Technology, 9585S, 1:1000), anti-SEMA6D (total cell lysates) (Abcam, ab191169, 1:250), and anti-SEMA6D (CM) (R&D, AF2095-SP, 1:1000). Following secondary antibody incubation, the detection was done using Vilber Fusion SL Imaging System.

### MTT Assay

6 ×10^4^ cells/well were seeded on a 24-well plate. Cells were treated with methylthiazolyldiphenyltetrazolium bromide (MTT) (Amresco, 0793) for 4 h on the second, fourth, sixth, and eighth days after plating. Following the addition of DMSO as the solvent, absorbance was read at 570 and 650 nm by using Thermo Multiskan Spectrum.

### Immunofluorescence and BrdU Assay

2.5 × 10^5^ MCF10A and 3.5 × 10^5^ MDA MB 231 and MCF7 cells/well were grown on coverslips on a 6-well plate for 48 h. For the BrdU assay, cells were incubated with 20 μM bromodeoxyuridine (BrdU) for 4 h. Cells were gently washed with PBS 1X and fixed with 1 mL of 4% Paraformaldehyde (PFA) in PBS 1X for 20 min at room temperature. Then the cells were permeabilized with 0.1% TritonX-100/PBS for 15 min and blocked with 5% BSA in 0.1% TritonX-100/PBS for 30 min at room temperature. Anti-BrdU (Cell Signaling Technology, 5292, 1:200) for BrdU, anti-V5 (CST, mAB13202, 1:1000) for V5 tag, and DAPI (Sigma, D9542, 1:500) for nucleus staining were used. Afterward, the cells were rinsed with PBS 1X (3 times, 10 min each) and mounted on Ibidi Mounting Medium (IMM, Cat. 50001). Fluorescent images were captured by using Olympus IX83 fluorescent microscope.

### Soft Agar Colony Formation Assay

3 ×10^4^ cells were mixed with 0.35% noble agar (BD Difco Noble Agar, 12185–010) and seeded on top of solidified 0.5% noble agar on 6-well plates. The complete medium was added after the solidification of the top layer, and the medium was changed twice a week for 6 weeks. After staining colonies with 0.05% crystal violet, the images focused on three different Z layers were captured for each well under a Leica DMI8 confocal microscope. The sizes of colonies that are higher than 30 μM in diameter were counted using ImageJ. Total colony numbers were normalized to the control condition in each of three independent experiments.

### Wound Healing Assay

7.5 × 10^5^ cells/well were seeded on a 12-well plate. The following day, cells were incubated with 10 μg/mL Mitomycin C (Santa Cruz, sc-3514A) for 2 h. Then the scratch was introduced with a 10 μL pipet tip, and 1% serum and 1% penicillin/streptomycin-containing medium were added. The gaps were monitored at 37 °C with 5% CO_2_ under a Leica DMI8 confocal microscope supplemented with the incubation chamber. Open area percentages were calculated for each position in three independent experiments.

### Invasion Assay

Three-channel lab-on-a-chip system (Initio) was used for invasion analysis, as explained previously.^17^ Briefly, cells were labeled transiently with a green fluorescent dye using Green Cell Tracker CMFDA (C2925, Invitrogen). The stock solution of Green Cell Tracker (25 mM) was diluted in serum-free DMEM to 5 μM final concentration. Following incubation with 5 μM CMFDA dye at 37 °C for 30 min, cells were washed with PBS and supplemented with the culture medium until loading. Then the growth factor reduced Matrigel (Corning, 356230) was mixed with the serum-free medium in a 1:1 ratio and loaded into the middle channel. After polymerization, 20% serum-containing medium was loaded to the lower channel, and labeled cells at the concentration of 1 × 10^6^ cells/ml in serum-free medium were loaded to the upper channel. The chips were incubated at 37 °C with 5% CO_2_ in a humidified incubator and visualized under Leica DMI8 confocal microscope for 3 days. Quantification was done as previously explained.^17^

### Data Mining Studies

UCSC Xena (http://xena.ucsc.edu/) was used to analyze mRNA expression of the SEMA6D gene in addition to cell cycle-related genes, epithelial-mesenchymal transition (EMT) genes, and Notch pathway genes (Table S2) using the TCGA database (https://www.cancer.gov/tcga) for breast-invasive carcinoma (1247 patients).^20^ The entire population was stratified based on SEMA6D mRNA expression quartiles, and the highest (0–25th %) Sema6D expressing samples were labeled as Sema6D-High and the lowest (75th %–100th %) Sema6D expressing samples were labeled as Sema6D-Low. Expression of the cell cycle, EMT, and Notch-related genes were compared between Sema6D-Low and -High populations. Results were presented as means ± SD. Data were analyzed for statistical significance by the Welch test (*t* test with unequal variances). Furthermore, cell cycle, EMT, and Notch-related genes were clustered based on uniform manifold approximation and projection (UMAP) for dimension reduction (arXiv:1802.03426) using BioVinci software and presented with respect to SEMA6D expression with a scoring threshold of 0.4.

### Statistical Analysis

A two-tailed, unpaired (samples with equal variance) Student’s *t* test method was used for statistical analysis unless stated otherwise.

## Results

### SEMA6D Has Opposing Effects on the Proliferation of Normal Breast Cell Line MCF10A and Breast Cancer Cell Line MCF7

**Figure 1:**
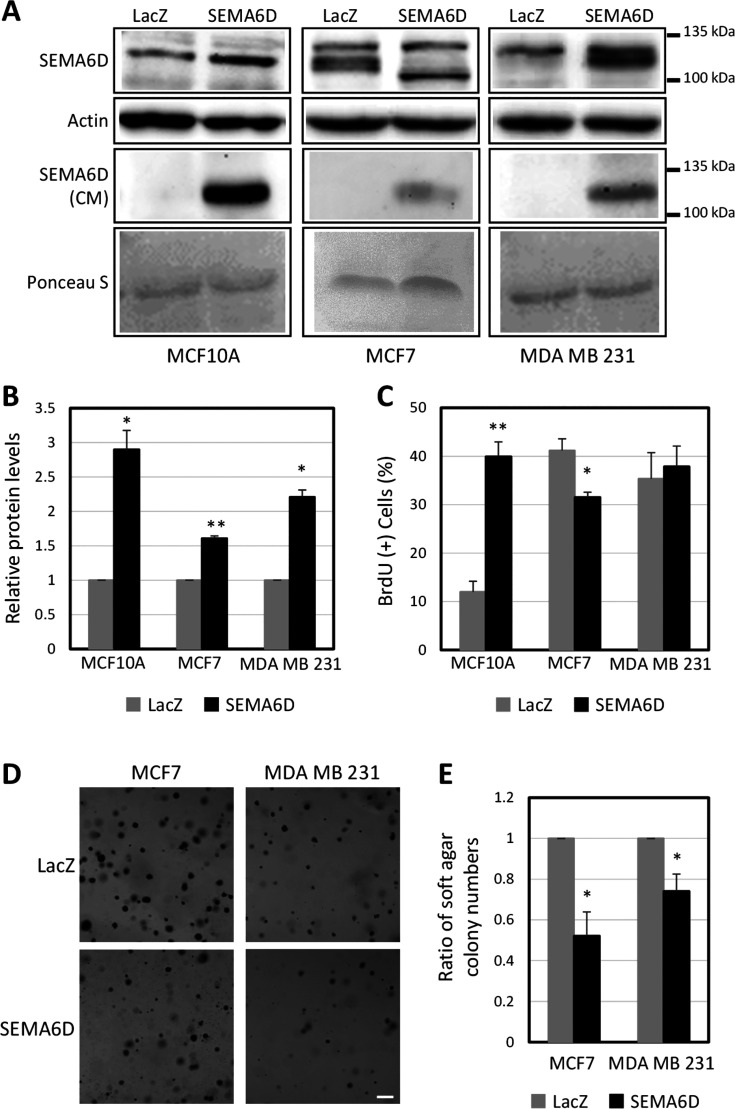
SEMA6D overexpression has opposing effects on proliferation and reduces anchorage-independent growth. (A) Representative Western blot images showing SEMA6D protein levels in total cell lysates (top) and in conditioned medium (CM) (bottom) of three cell lines that overexpress LacZ or SEMA6D. (B) Quantification of SEMA6D protein levels in total cell lysates. (C) Ratio of BrdU incorporation for MCF10A, MCF7, and MDA MB 231 cell lines that overexpress LacZ or SEMA6D. (D) Representative images of soft agar colonies formed by MCF7 and MDA MB 231 cell lines that overexpress LacZ as control or SEMA6D (scale bar: 200 μm). (E) Ratio of soft agar colony numbers. Data is represented as mean ± SD of two independent experiments. (*p < 0.05, **p < 0.005).

First, we generated stable cell lines that overexpress SEMA6D to investigate its role in breast cells. We used MCF7 and MDA MB 231 as breast cancer cell lines, and MCF10A as a normal cell line. SEMA6D mRNA expression increased more than 600-fold in all cell lines compared to control cells, which stably expressed LacZ (Figure S1A). In accordance with previous results, a higher SEMA6D protein signal was detected in total cell lysates (Figure 1A, top panel). Protein levels were increased by 2.9- and 2.2-fold upon stable overexpression in MCF10A and MDA MB 231 cells, respectively (Figure 1B). Although we detected a 1.6-fold significant increase at the expected size range for MCF7 cells, because of the presence of several bands between LacZ and SEMA6D samples, we used an additional approach using the V5 tag at the C-terminal of the inserts for confirmation. Immunofluorescence assay showed that MCF7 cells that overexpress LacZ or SEMA6D are positive V5 signal (Figure S1B). Furthermore, we detected SEMA6D in the conditioned media of all the cells that overexpress SEMA6D (Figure 1A, bottom panel), confirming proteolytic processing of the protein as observed previously in different tissues.^5,8,11^

Next, we analyzed whether SEMA6D overexpression affects the proliferation rate of the cells by BrdU incorporation assay. SEMA6D overexpression increased BrdU incorporation from 11.9% to 39.9% in MCF10A cells (Figure 1C). On the other hand, in MCF7 cells the ratio of BrdU positive cells was decreased from 41.2% to 31.6% in response to SEMA6D overexpression (Figure 1C). To assess whether the change in proliferation rate is reflected in the growth pattern, we analyzed the growth curve of the cells by MTT assay. In parallel with the BrdU results, MCF10A cells that overexpress SEMA6D reached higher numbers earlier than the control cells that overexpress LacZ. Eight days after plating an equal number of cells, the number of total alive cells in MCF10A-SEMA6D and MCF10A-LacZ groups were increased by 20.5 and 16.5-fold, respectively (Figure S2). Consistent with the decrease in BrdU incorporation in response to SEMA6D overexpression, the number of alive cells in the MCF7-LacZ group was increased by 11.9-fold, which was reduced to 9.9-fold in MCF7-SEMA6D group (Figure S2). In MDA MB 231 cells, SEMA6D overexpression did not change either the growth curve pattern or the BrdU incorporation rate (Figure 1C and S2).

Overall, these data showed that SEMA6D overexpression has variable effects in different cell lines that it induces proliferation in nontumorigenic MCF10A cells while reducing it in MCF7 cancer cells and showed no effect on MDA MB 231 cancer cells.

### SEMA6D Reduces Anchorage-Independent Growth Potential of Breast Cancer Cell Lines

Anchorage-independent growth is correlated with the tumorigenic potential of the cancer cells. Thus, we analyzed how SEMA6D affects anchorage-independent growth potential using soft agar assay. SEMA6D overexpression reduced the number of colonies formed in soft agar both for MCF7 and MDA MB 231 cells (Figure 1D). Specifically, the decrease was 48% for MCF7 and 26% for MDA MB 231 cells (Figure 1E). MCF10A cells did not form any colonies in either condition, indicating that SEMA6D overexpression cannot induce tumorigenicity in the normal breast cells. On the contrary, SEMA6D reduces the tumorigenic potential of breast cancer cells.

### SEMA6D Induces Migration and Invasion of Breast Cell Lines

**Figure 2:**
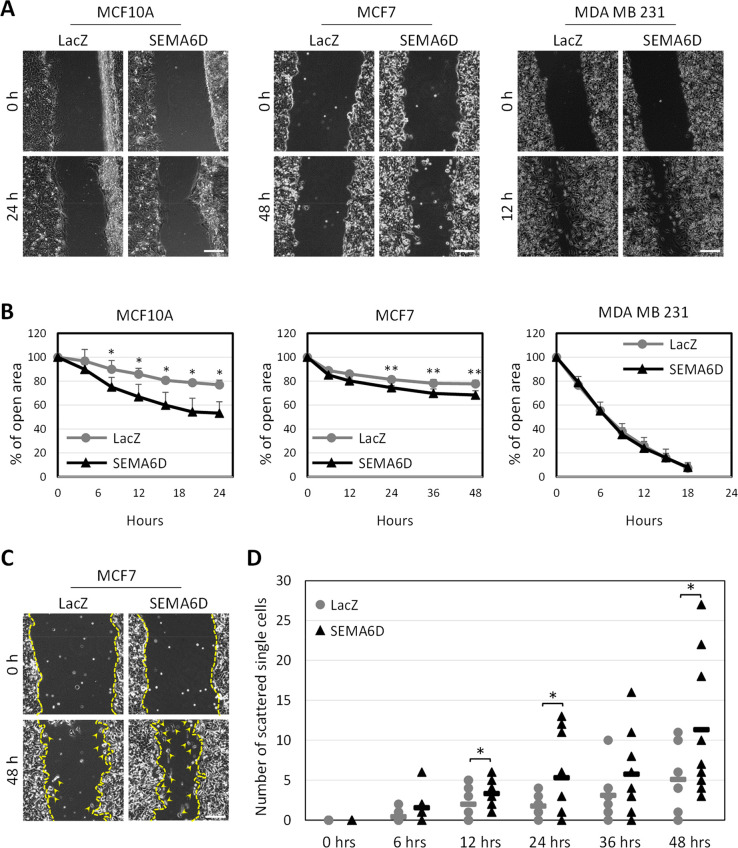
SEMA6D increases migratory potential. (A) Representative images of the cell lines that overexpress LacZ as control or SEMA6D at 0, 24 (MCF10A), 48 (MCF7), and 12 h(MDA MB 231) after scratching are shown. (B) Percentage of open area for each condition is plotted for different time points after scratching. Data is represented as mean ± SD of three independent experiments. (C) Representative images of MCF7 cell lines at 0 and 48 h after scratching are shown. Yellow dotted lines represent the border drawn for wound healing analysis. Arrowheads show the scattered single cells detached from the border cells. (D) Number of scattered single cells at different time points after scratching are shown for MCF7. Each dot represents one image analyzed, and the line segment represents the average of three independent experiments. (*p < 0.05, **p < 0.005) (scale bar: 100 μm).

Migration and invasion potential of the cancer cells are closely related to the aggressive phenotype of tumors. Thus, we analyzed whether SEMA6D has an effect on migration and invasion. Migration potential was assessed by wound healing assay, in which MCF10A and MCF7 cells that overexpress SEMA6D covered a larger area compared to the control cells 24 and 48 h after scratching, respectively (Figure 2A). The image analysis showed that SEMA6D overexpression reduced the percentage of the open area to 53.2% in MCF10A cells compared to control cells, where the open area was 76.9% at 24 h (Figure 2B). Similarly, in MCF7 cells that overexpress SEMA6D, open area was reduced to 68.4%, which was 77.7% in the control group at the end of 48 h (Figure 2B). Furthermore, SEMA6D overexpression changed the migration pattern of MCF7 cells. We observed a higher number of scattered single cells dissociated from the wound border in the case of SEMA6D overexpression (Figure 2C). On average, there were 3.3, 5.3, and 11.3 dissociated cells in MCF7-SEMA6D compared to 2, 1.8, and 5.1 cells in the control MCF7-LacZ cells at 12, 24, and 48 h after scratching, respectively (Figure 2D). In MDA MB 231 cells, we did not observe any effect of SEMA6D overexpression on the migration potential or pattern of the cells (Figure 2A and B).

**Figure 3:**
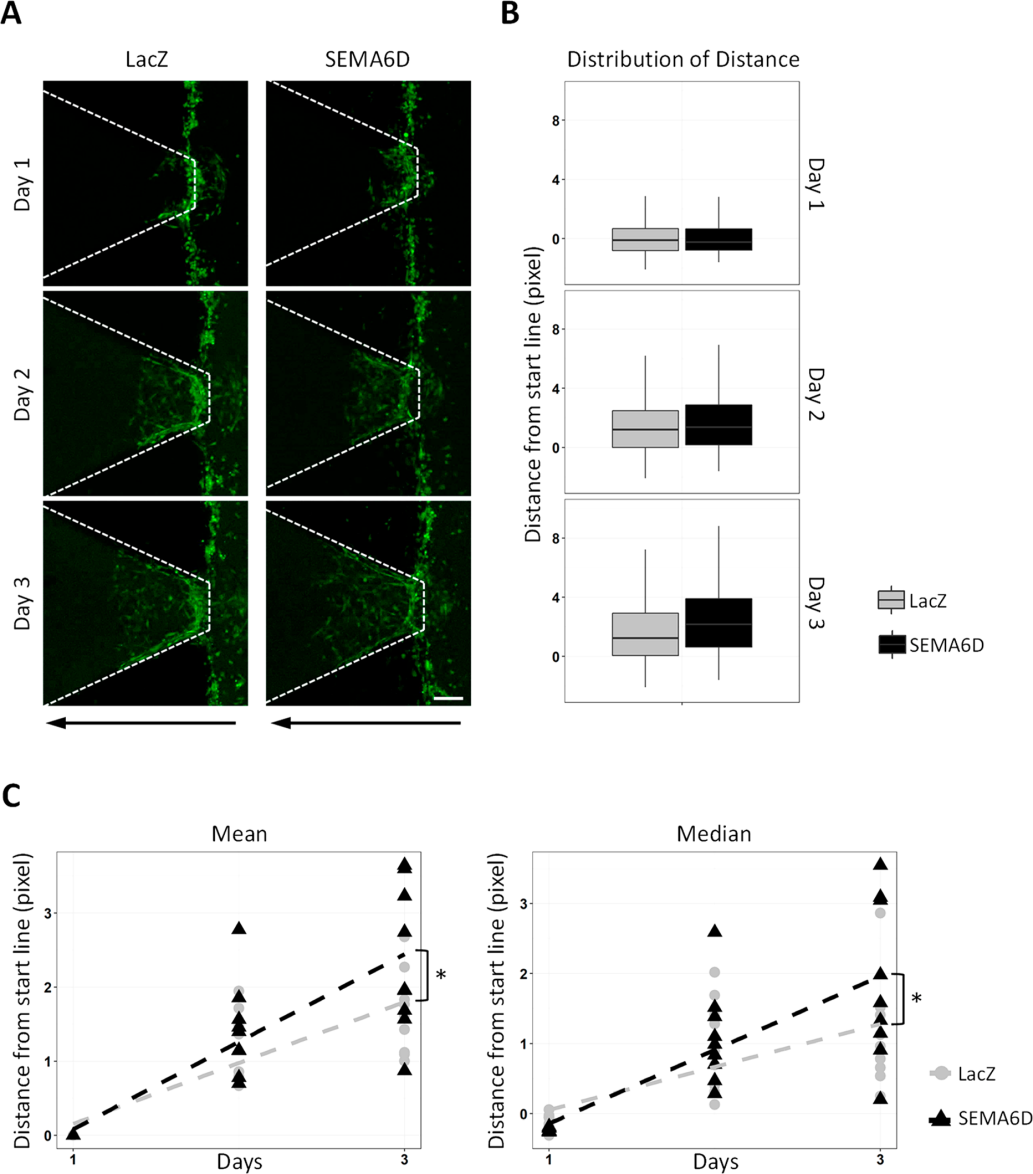
SEMA6D promotes invasion of MDA MB 231 cells. (A) Representative Z-stack images of green-labeled MDA MB 231 cells stably expressing LacZ or SEMA6D on day 1, 2, or 3 after loading on invasion chips are shown. Dashed lines mark the Matrigel channel. Vertical dashed lines show gates where cells can pass through. Black arrows show the direction of invasion (scale bar: 100 μm). (B) Following thresholding of the Z-stack images, the distance of each bright pixel to starting line (vertical dashed line in A) was calculated and normalized to day 1 for each gate. All of the distance values for nine gates from three independent experiments were plotted. (C) Mean and median distance values of three independent experiments were plotted. Each dot represents one gate (*p < 0.05).

For the evaluation of the invasion, we used a three-channel lab-on-a-chip (LOC) system in which cells labeled with green fluorescent dye invade through Matrigel loaded channel toward serum-rich medium.^17^ On each LOC, the Matrigel channel is separated from the cell channel by three gates, through which cells receive chemoattractant signals and invade Matrigel. Invasion of MDA MB 231 cells that overexpress LacZ or SEMA6D was observed for 3 days (Figure 3A). The distribution of the distance invaded by the cells that overexpress SEMA6D was shifted toward higher values compared to control cells that overexpress LacZ (Figure 3B). Moreover, SEMA6D overexpression increased the mean and the median distance invaded by the cells (Figure 3C). On the other hand, SEMA6D overexpression did not induce an invasive behavior in MCF7 and MCF10A cells (data not shown).

Taken together, these results demonstrate that SEMA6D induces migration in the normal cell line MCF10A and the cancer cell line MCF7, whereas although it cannot trigger a migratory phenotype per se, SEMA6D can augment the invasive potential of MDA MB 231 cancer cells.

### SEMA6D Affects the Expression of Epithelial and Mesenchymal Markers

**Figure 4:**
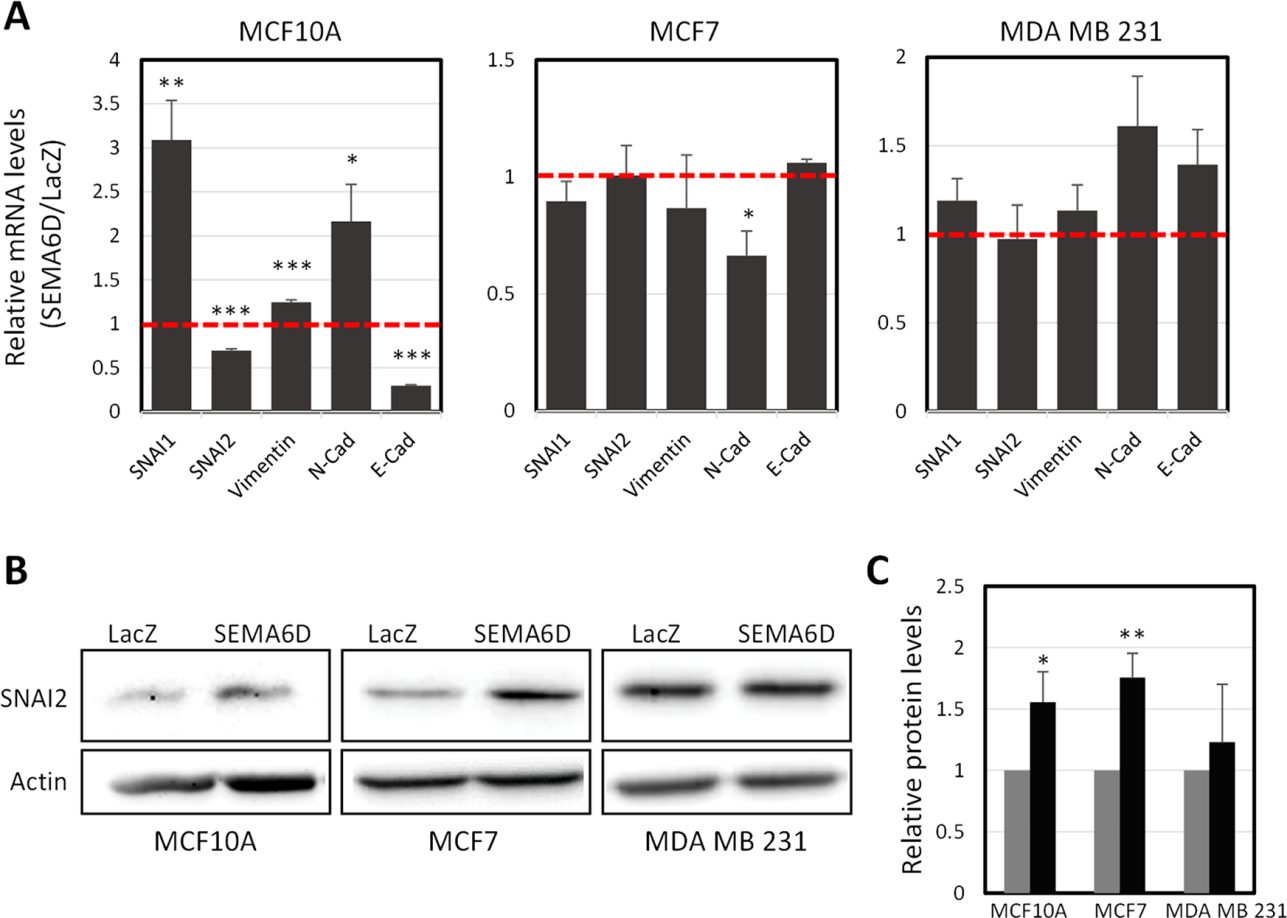
SEMA6D overexpression induces the expression of mesenchymal markers. (A) Ratio of mRNA expressions of mesenchymal (SNAI1, SNAI2, vimentin, and *N*-cadherin) and epithelial (*E*-cadherin) markers in the SEMA6D overexpressing group compared to the LacZ overexpressing group are shown. The dashed line represents the relative expression level in the LacZ group. Data is represented as the mean ± SD of three independent experiments. (B) Representative Western blot images and (C) quantification of SNAI2 and *N*-cadherin protein levels are shown for the three cell lines that overexpress LacZ (gray) or SEMA6D (black). Data is represented as mean ± SD of two independent experiments. (*p < 0.05, **p < 0.005, ***p < 0.0001).

Increased migration rate in MCF10A and MCF7 cells, single-cell migratory pattern of MCF7 cells, and increased invasion of MDA MB 231 cells in response to SEMA6D overexpression point to a more mesenchymal phenotype. Thus, we analyzed the expression pattern of epithelial-mesenchymal-transition (EMT) markers to investigate whether SEMA6D overexpression affects the EMT phenotype. In MCF10A cells, mRNA expression of mesenchymal markers SNAI1, vimentin, and *N*-cadherin was increased, while mesenchymal marker SNAI2 and epithelial marker *E*-cadherin was downregulated upon SEMA6D overexpression (Figure 4A). In the MCF7 cells that overexpress SEMA6D, mesenchymal marker *N*-cadherin was significantly reduced compared to control cells. Other than that, SEMA6D overexpression did not significantly affect mRNA levels of the EMT markers in MCF7 and MDA MB 231 cells (Figure 4A).

SNAI2 is one of the main regulators of EMT in breast cancer^21^ and was previously shown to be correlated with SEMA6D in breast cancer;^15^ thus, we analyzed SNAI2 protein (Slug) expression (Figure 4B). SEMA6D overexpression increased SNAI2 protein by 1.6- and 1.8-fold in MCF10A and MCF7 cells, respectively (Figure 4C). In MDA MB 231 cells, we did not observe any significant difference in SNAI2 (Figure 4B,C).

Overall, these results suggest that SEMA6D could induce a mesenchymal phenotype via upregulation of SNAI2 in epithelial MCF10A and MCF7 cells, while it has no effect on EMT in MDA MB 231 cells, which has a more mesenchymal phenotype, to begin with.

### Differential Expression of SEMA6D in Invasive Breast Carcinoma Is Coupled with Differential Expression of Cell Cycle Regulators and Epithelial-Mesenchymal Transition Markers in Human Breast Tumors

Finally, we analyzed whether the effects of SEMA6D overexpression we demonstrated in the cell lines had clinical relevance in breast cancer using The Cancer Genome Atlas (TCGA) data. For this purpose, we sorted all samples with respect to SEMA6D expression and selected the highest expressing quartile (SEMA6D-High, *n* = 273) and the lowest expressing quartile (SEMA6D-Low, *n* = 273) as subgroups. Compared to SEMA6D-Low samples, SEMA6D-High samples had 81% higher SEMA6D gene expression (Figure S3A). mRNA expressions of cell cycle-related genes were mostly downregulated in the SEMA6D-High group compared to SEMA6D-Low samples (Figure S3B, with the highest suppression in CCNE1 (−25.9%, *p* < 0.001)). The UMAP algorithm reduced the dimension of cell cycle genes in six clusters based on expressions (Figure S3C).

**Figure 5:**
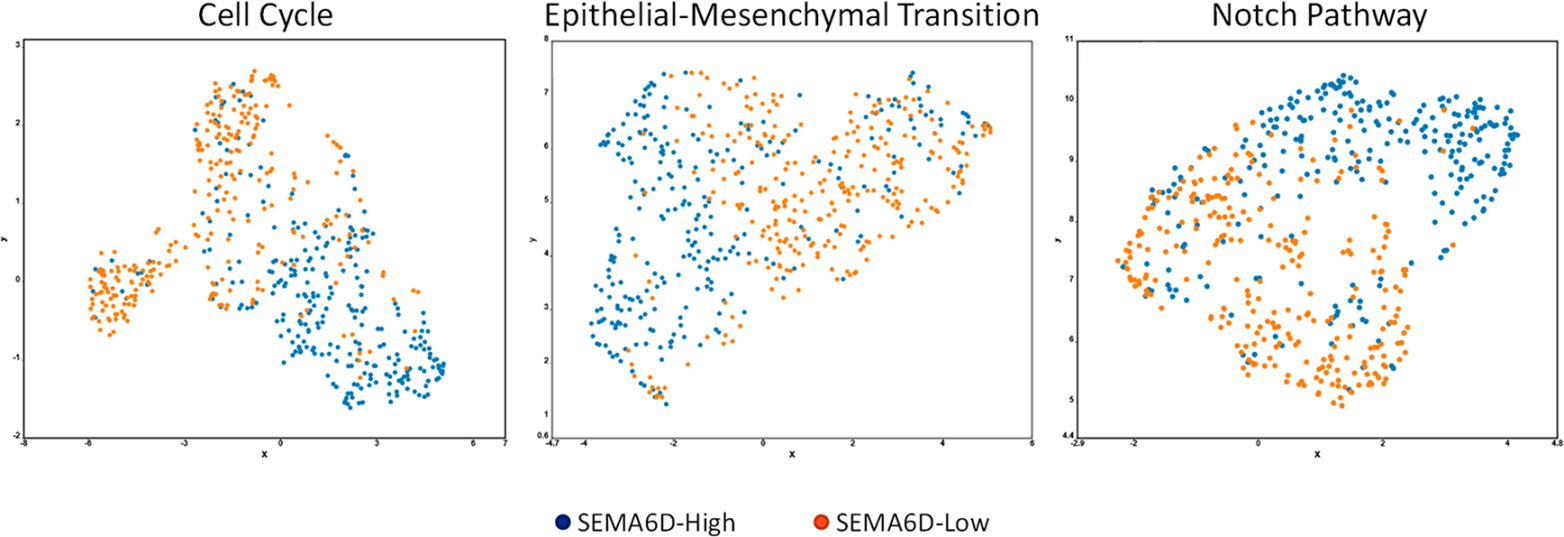
SEMA6D-High and SEMA6D-Low subgroups of invasive breast carcinoma are represented on gene-expression-based clusters. SEMA6D-High (*n* = 273) and SEMA6D-Low (*n* = 273) samples are represented on reduced dimension plots based on expressions of the cell cycle, epithelial-mesenchymal transition, and Notch pathway-related genes.

Comparisons based on SEMA6D expression status demonstrated a clear distinction between SEMA6D-High and SEMA6D-Low samples, with downregulation of E2F1, E2F2, CDC20, CCNB1, CCNB2, CDC25A, PCNA, CDK1, CDC25C, CDC6, CCNE1, and CDC25B in the SEMA6D-High group (cut of scores >0.4, sorted from highest to lowest score) (Figure 5). Genes related to EMT were also affected by the SEMA6D status (Figure S3B). CDH1 (−5%) and SNAI1 (−4%) expressions were significantly lower in SEMA6D-High samples, while the remaining EMT markers showed significantly higher expressions. The UMAP algorithm detected nine clusters for EMT marker genes (Figure S3D). Still, once this dimensionally reduced expression data was stratified based on SEMA6D status, SEMA6D-Low samples showed a clear separation from the SEMA6D-High group based on downregulation of ZEB1, ZEB2, and SNAI2 genes with cutoff values of 0.95, 0.58, and 0.44, respectively (Figure 5).

Notch signaling is one of the signaling pathways that is deregulated in breast tumorigenesis. We and others have shown that in addition to affecting proliferation and transformation, Notch activation also induces EMT in breast tissue.^17^ Thus, we later asked whether SEMA6D status is associated with Notch signaling. The majority of the genes related to Notch signaling were significantly upregulated in SEMA6D-High samples, except DLL3, which had a 47% (*p* < 0.001) reduction (Figure S3B). UMAP dimensional reduction identified seven clusters (Figure S3E); however, stratification based on SEMA6D status showed a distinction based on NOTCH4 and DLL4 upregulation, with the cutoff values of 0.94 and 0.53, in SEMA6D-High samples (Figure 5). Next, we analyzed whether Notch signaling activity might be involved in SEMA6D regulation via activating the pathway upon overexpression of Notch1 intracellular domain (NICD) in MCF10A cells. SEMA6D mRNA (Figure S4A) and protein (Figure S4B and C) expressions were upregulated in response to Notch activation suggesting the Notch pathway as a potential upstream regulator of SEMA6D.

## Discussion

The greater part of the research on SEMA6D investigated its role in developmental processes, while the comparatively small number of studies explored whether and how SEMA6D is involved in tumorigenesis. Specifically, the two studies focusing on SEMA6D in breast cancer are limited to the analysis of expression and mutation data of breast cancer patients and suffer from the lack of experimental evidence.^15,16^ Thus, the aim of this study was to develop a better understanding of how SEMA6D affects the molecular and cellular properties of normal breast and breast cancer cell lines.

First, we investigated the effect of SEMA6D overexpression on proliferation. Prior studies have not established a consistent association between SEMA6D and proliferation. In different regions of the developing heart, SEMA6D was shown to regulate the migration of mesenchymal and endothelial cells but had no effect on the proliferation.^5,8^ On the other hand, a more recent study noted that proliferation of prenatal cardiomyocytes was reduced in SEMA6D knockout mouse model, which also had less Cyclin D1 and Cyclin D2 levels.^7^ In line with this result, SEMA6D overexpression was reported to induce proliferation in osteosarcoma cell line HOS, which contributes to its oncogenic role.^9^ In accordance with the previous observations, our results also demonstrate a cell-specific effect of SEMA6D on the proliferation of breast cells. SEMA6D overexpression induced proliferation in the normal breast cell line, MCF10A, hinting at a tumor-initiating role. However, the inability to induce anchorage-independent growth of MCF10A cells indicates that SEMA6D per se could not act as a strong oncogene activating these two traits simultaneously in nontumorigenic breast cells. On the other hand, SEMA6D overexpression reduced proliferation in MCF7 cells, illustrating an antitumorigenic role, which is also supported by reduced anchorage-independent growth of both breast cancer cell lines. Our results differ from the positive correlation demonstrated between SEMA6D expression and anchorage-independent growth in malignant mesothelioma and osteosarcoma cells^9,11^ highlighting the importance of tissue and cell context in tumorigenesis. In accordance with the experimental data, TCGA analysis revealed an association between SEMA6D levels (high vs low) and the expression of cell cycle-related genes. Although CCND2 and CCND1 were among the upregulated genes in the SEMA6D-High subpopulation, the majority of the positive regulators of the cell cycle, including CCNE1, CDC25C, CDC20, E2F1, and E2F2, were downregulated at a greater magnitude, which might reflect the growth-inhibitory effect of SEMA6D in MCF7 cells. Whether increased proliferation due to SEMA6D overexpression observed in MCF10A cells represents an early stage of breast tumorigenesis remains unknown due to the lack of relevant clinical sample group in the data set.

A number of studies described a link between SEMA6D and migration mainly during embryonic development. SEMA6D was demonstrated as an essential molecule involved in cardiac development and neural tube closure, during which its major effect was on the migration of mesenchymal and endothelial cells.^8^ Likewise, BMP-induced migration during atrioventricular cushion development was dependent on SEMA6D upregulation.^5^ SEMA6D was reported as a repulsive regulator for dorsal root ganglion, the crossing of retinal ganglion cells, and pericyte–endothelial cell interaction, all of which are related to migratory behavior of cells.^3,22^ A recent study that focused on osteosarcoma noted that SEMA6D overexpression rescued the migration and invasion potential, which was decreased in response to knockdown of cicUBAP2, a positive regulator of SEMA6D expression.^10^ Here, we showed that SEMA6D induces migration in MCF10A and MCF7 cell lines but does not affect MDA MB 231 cells. Considering that the latter could be explained by the extensively migratory nature, we also analyzed invasion and demonstrated that SEMA6D promoted the invasion potential of MDA MB 231 cells. Together, in addition to confirming the previous findings, these results presented the first evidence that in breast cells, SEMA6D can promote migration and invasion.

Although SEMA6D was implicated in migration in the context of development and cancer, its role in EMT, which is related to the migratory phenotype, has not been investigated thoroughly. In breast cancer, TCGA analysis showed that the SEMA6D-high patient group has decreased *E*-cadherin and increased SNAI2, ZEB1, and ZEB2, suggesting a correlation between SEMA6D and mesenchymal transition.^15^ In gastric cancer, colocalization of SEMA6D with SNAI1 protein (Snail) was positively correlated with invasion and lymph node metastasis.^23^ Here, we showed that SEMA6D regulated mRNA expression of the EMT markers, mainly shifting toward a more mesenchymal pattern in MCF10A cells. On the other hand, in breast cancer cells, there was no change in the mRNA levels of EMT markers other than a decrease in *N*-cadherin in MCF7, which was not anticipated considering the shift toward a more migratory and invasive phenotype in response to SEMA6D overexpression. On the other hand, SNAI2 (Slug) protein was increased in MCF7 cells, consistent with a more mesenchymal phenotype. Supporting the experimental data, SEMA6D levels were associated with the expression of EMT markers in the TCGA data set. Consistent with the previous study,^15^ in the SEMA6D-High group mesenchymal markers, including ZEB1, SNAI2, ZEB2, and TWIST, were increased. Collectively, the data we present here suggest a correlation between SEMA6D expression and a migratory/invasive phenotype supported by a mesenchymal shift in the EMT markers’ expression, which are in accordance with the previous studies. Although both our experimental data and TCGA data analyzed by us and others converge on SNAI2 in focus, the existing data fail to provide clear evidence of a direct transcriptional and translational regulation of EMT by SEMA6D.

SEMA6D induced a similar trend in breast cancer cell lines, such as a decrease in anchorage-independent growth and an increase in mobility. However, the two cell lines showed distinct phenotypes in migration and expression profile of EMT markers. Although the cell lines used in this study represent the characteristic properties of luminal (MCF7) and basal (MDA MB 231) breast cancer subtypes, the data cannot be extrapolated to subtype-specific roles of SEMA6D. This issue needs to be explored in further research using a panel of cell lines representing different subtypes.

Finally, we showed an association between Notch pathway-related genes and SEMA6D levels in the TCGA data set and induction of SEMA6D expression upon Notch activation in MCF10A cells. Although our data suggest that Notch signaling might be an upstream regulator of SEMA6D in the breast, the functional importance of Notch-SEMA6D relation in breast tumorigenesis remains to be answered.

In summary, here, we showed a cell-specific effect of SEMA6D in normal and tumorigenic cell lines of the breast. A possible explanation for this observation is the different downstream mechanisms activated upon SEMA6D overexpression in different cell lines. A notable example in this context comes from osteosarcoma, where only two out of four cell lines had increased ERK phosphorylation to different extents upon SEMA6D overexpression.^9^ Although Plexin A1 was reported as the receptor of SEMA6D, other cell-surface proteins such as vascular endothelial growth factor receptor 2 (VEGFR2), Off-Track (OTK), Ng-CAM related cell adhesion molecule (Nr-CAM), and Plexin A4 were also shown to interact with SEMA6D.^2,3,8,11^ Thus, differential representation of SEMA6D interacting proteins on the cell surface could also explain cell-specific effects that we observed. Another reason for the cell-specific effect could be the endogenous expression profile of SEMA6D. Although we observed a single band in the conditioned media, different bands of SEMA6D were visible in whole cell lysates of each cell line, which might indicate distinct endogenous SEMA6D isoforms. As SEMA6D has nine isoforms,^6,24^ we cannot exclude the possibility of SEMA6D overexpression interfering with endogenous SEMA6D isoforms and contributing to the manifestation of different phenotypes. Although the issue of different isoforms is an intriguing one, to date there is no data on the functional importance of different SEMA6D isoforms in breast cancer, and it requires further research.

In this study, we provided the first experimental evidence on the functional role of SEMA6D in normal and tumorigenic breast cell lines. However, there is an abundant room for further investigations to answer the questions such as interacting partners on the cell surface, upstream regulators, and downstream mediators of SEMA6D to develop a full picture of SEMA6D’s role in breast cancer.

## Supporting Information Available

Tables of primer sequences and gene lists used in bioinformatics analysis; figures showing expression levels of SEMA6D mRNA and growth curve of cell lines in response to overexpression of SEMA6D; figure showing gene-expression profiles of SEMA6D-High and -Low subgroups of breast carcinoma; figure showing SEMA6D expression levels in response to activation of Notch signaling (PDF)

## Notes

**Compliance with Ethical Standards.** The authors declare that principles of ethical and professional conduct have been followed. The sources of funding and potential conflicts of interest are declared. The research does not involve human participants and/or animals.

## Notes

**Availability of Data and Material.** The data and material presented in this study are available from the corresponding author upon request.

## Notes

TOC/Abstract graphic created with BioRender.com.
