# Supplementary material for: SEMA6D Differentially Regulates Proliferation, Migration, and Invasion of Breast Cell Lines
Source: ACS Omega. 2022 Apr 27;7(18):15769–78. doi: 10.1021/acsomega.2c00840 (PMC9097209; doi:10.1021/acsomega.2c00840)
Supplement: Supplementary file 1 — ao2c00840_si_001.pdf [file ao2c00840_si_001.pdf]

SUPPORTING INFORMATION

SEMA6D differentially regulates proliferation, migration, and invasion of breast cell lines

Zehra Elif Gunyuz[a], Ece Sahi-Ilhan[a], Cansu Kucukkose[a], Dogac Ipekgil[a], Gunes Tok[a], Gulistan Mese[a], Engin Ozcivici[b], Ozden Yalcin-Ozuysal[a, *]

Affiliations:

[1] Department of Molecular Biology and Genetics, Izmir Institute of Technology, 35430, Izmir, Turkey

[2] Department of Bioengineering, Izmir Institute of Technology, 35430, Izmir, Turkey

Corresponding Author:

Ozden Yalcin-Ozuysal, PhD

ozdenyalcin@iyte.edu.tr

Table S1. Primer Sequences

Gene	Forward Primer (5' -3')	Reverse Primer (5' -3')
**E-Cadherin**	CAGCACGTACACAGCCCTAA	GGTATGGGGGCGTTGTCATT
**N-Cadherin**	CCTCCAGAGTTTACTGCCATGAC	GTAGGATCTCCGCCACTGATTC
**SEMA6D**	TTTCCCAGTTGAGGGCAGTC	AGGGCGTCCTCTAAAAACCG
**SNAI1**	CTAGGCCCTGGCTGCTACAA	TGTGGAGCAGGGACATTCG
**SNAI2**	CTCCTCATCTTTGGGGCGAG	TTCAATGGCATGGGGGTCTG
**TBP**	TAGAAGGCCTTGTGCTCACC	TCTGCTCTGACTTTAGCACCTG

Table S2. Gene Lists

**Cell Cycle Related Genes**	CCND1	CCNE1	E2F3	MDM2	MKI67	CDK2
	CDK4	CDK6	CDK7	E2F1	E2F2	E2F4
	E2F5	CDC26	ANAPC10	ANAPC13	ANAPC2	ANAPC4
	ANAPC5	ANAPC7	ANAPC11	PCNA	ANAPC1	CCNA1
	CCNB1	CCND2	CCND3	CCNB2	CCNE2	CDK1
	CDC6	CDC20	CDC25A	CDC25B	CDC25C	CDKN1A
	CDKN2A	TP53	ATM	FBXW7	RB1	CDKN1B
	CDKN1C	CDKN2A	CDKN2B	CDKN2C	CDKN2D	
**Epithelial-Mesenchymal Transition Related Genes**	CDH1	CDH2	SNAI1	SNAI2	VIM	ZEB1
	ZEB2	TWIST1				
**Notch Pathway Related Genes**	NOTCH1	NOTCH2	NOTCH3	NOTCH4	JAG2	JAG1
	DLL1	DLL3	DLL4	MAML1	MAML2	MAML3
	NUMB	HES1	HES5	HEY1	HEY2	

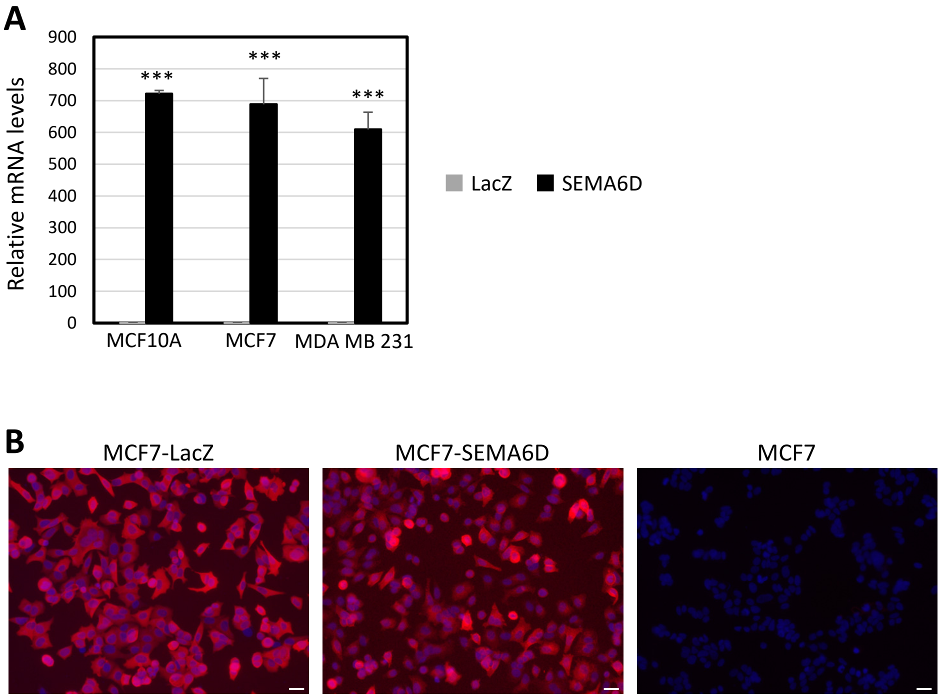

Figure S1. SEMA6D levels are increased upon overexpression in breast cell lines. (A) Relative mRNA expression of SEMA6D is shown for MCF10A, MCF7 and MDA MB 231 cell lines that overexpress LacZ as control or SEMA6D. Data is represented as mean±S.D. of three independent experiments. (*** p<0.0005) **(B)** V5 expression is detected by immunofluorescence in MCF7 cells that overexpress LacZ as control or SEMA6D. Red is for V5 and blue represents nucleus. (Scale bar: 50 µm)

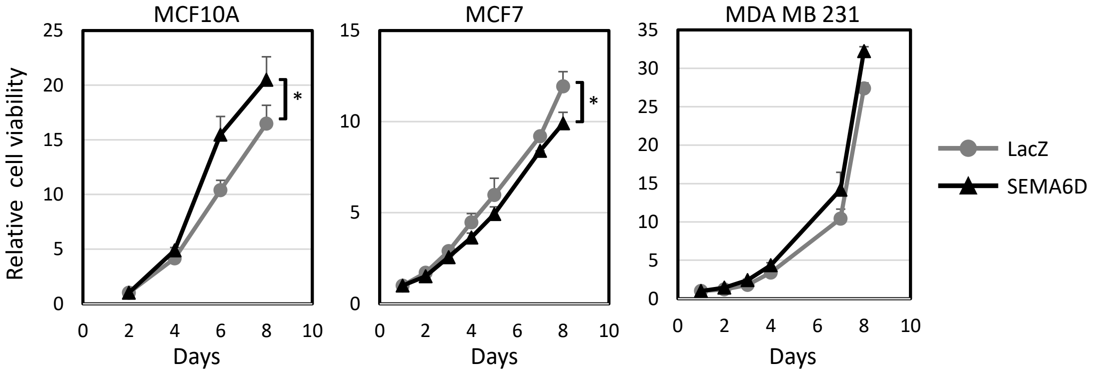

Figure S2. Effects of SEMA6D overexpression on growth curves. Relative cell viability for three cell lines that overexpress LacZ or SEMA6D is quantified for 8 days by MTT assay. Data is represented as mean±S.D. of three independent experiments. (* $p<0.05$)

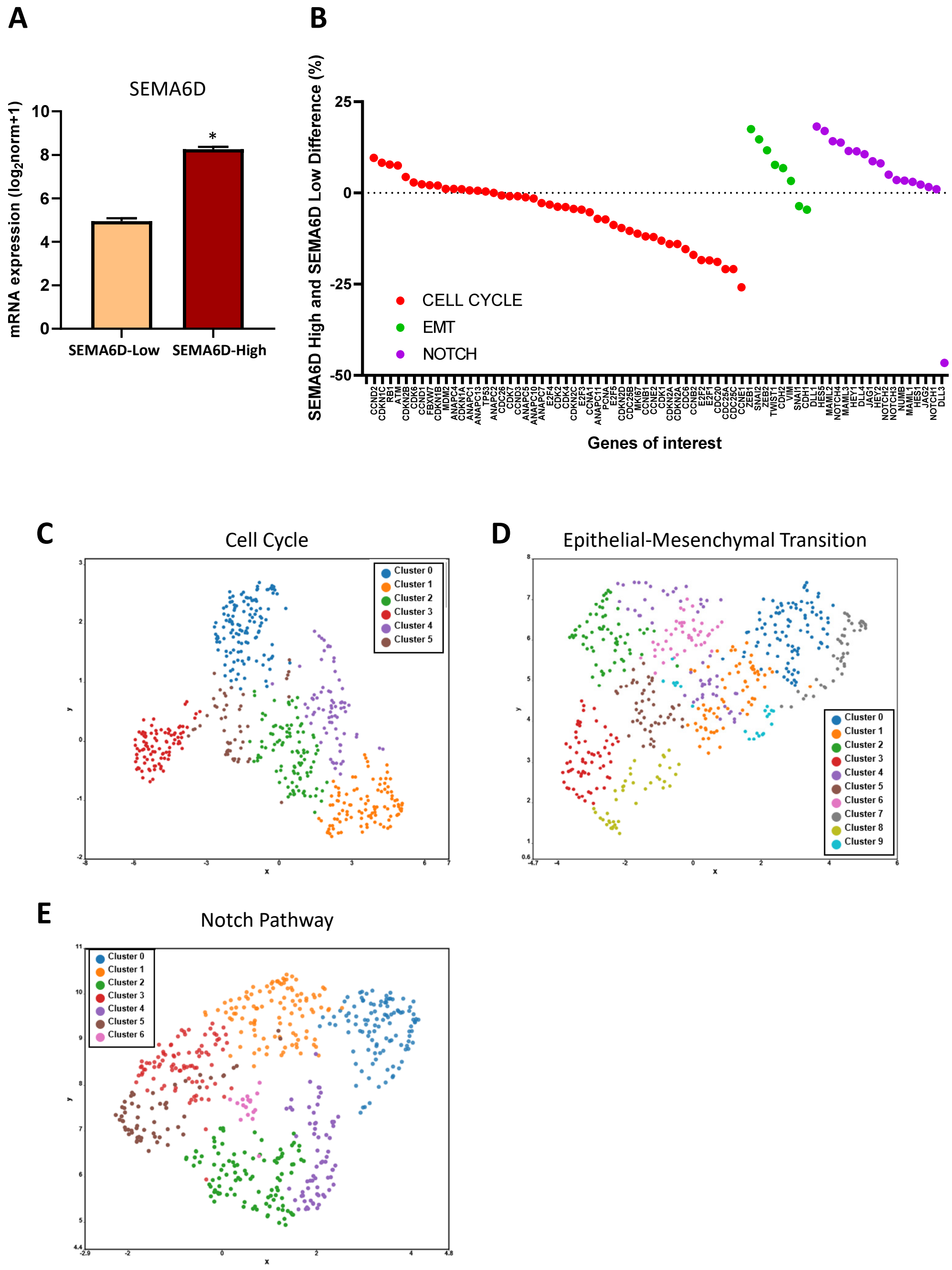

Figure S3. Gene expression profile of SEMA6D-High and SEMA6D-Low subgroups of invasive breast carcinoma. (A) SEMA6D expression levels in SEMA6D-High (n=273) and SEMA6D-Low (n=273) groups of invasive breast carcinoma samples from TCGA. **(B)** Difference in gene expression levels in SEMA6D-High subgroup compared to SEMA6D-Low subgroup represented in percentile. UMAP plots of dimensional reduction for the entire dataset based on expressions of **(C)** cell cycle, **(D)** epithelial-mesenchymal transition and **(E)** Notch pathway related-genes. (* p<0.001)

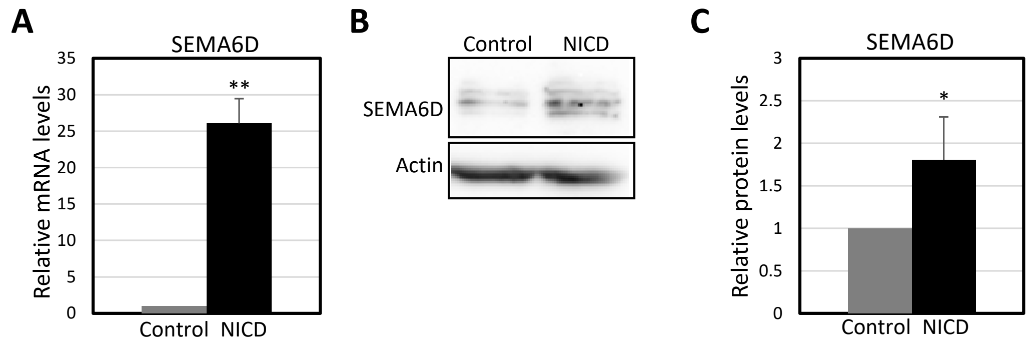

Figure S4. SEMA6D expression in response to Notch activation. (A) Relative mRNA expression of SEMA6D in MCF10A cells infected with a retrovirus expressing active Notch1 receptor (NICD) or control virus. **(B)** Representative Western blot images and **(C)** quantification of SEMA6D protein levels in control cells or cells with NICD overexpression. Data is represented as mean±S.D. of three independent experiments. (* $p<0.05$, ** $p<0.005$)
